# The controversial role of glucose in the diabetic kidney

**DOI:** 10.1097/j.pbj.0000000000000113

**Published:** 2021-01-26

**Authors:** Rui Fernandes

**Affiliations:** Instituto de Inovação e Investigação na Saúde – i3S, Universidade do Porto, Porto, Portugal

**Keywords:** glycogen, renal gluconeogenesis, renal glucose metabolism

## Abstract

The kidneys play an important role in maintaining glucose homeostasis being the main mechanisms, the gluconeogenesis, renal glucose consumption and glucose reabsorption in the proximal tubules. In this review, we present the main research into the role of glycogen—the stored form of glucose, and how it accumulates in the cells, providing new information on the link between diabetes and diabetic kidney disease. In the last 10 years, research under the scope of renal insulin handling, glucose transport in the proximal tubules, renal gluconeogenesis and renal insulin resistance, made possible to relate the roles of glucose and glycogen in the kidney with other several organs, like the liver. On the one hand, insulin positively regulates kidney uptake and degradation, and there is probably a specific action and resistance to insulin at the renal site. Moreover, insulin regulates the bioavailability of the sodium-glucose co-transporters—SGLT2 inhibitor, and inhibits renal gluconeogenesis. Only the liver and kidneys can supply glucose to the circulation through the process of gluconeogenesis, which involves the synthesis of glucose again from non-glycemic substrates; and the decomposition of stored glycogen. In the mind of nephrologists, diabetologists and scientists, glucose metabolism in the kidney is the focus, with the relevant success of inhibitors in reducing kidney and cardiovascular diseases in individuals with diabetes. However, these new data led to the intriguing paradigm that many of the beneficial effects on the renal and cardiovascular system appear to be independent of the systemic glucose-lowering actions of these agents. The goal of this work puts in context a highly relevant research area for renal glucose metabolism, of glycogen accumulation and metabolism in the diabetic kidney.

## Introduction of Glucose to Metabolic Syndrome

**Figure 1:**
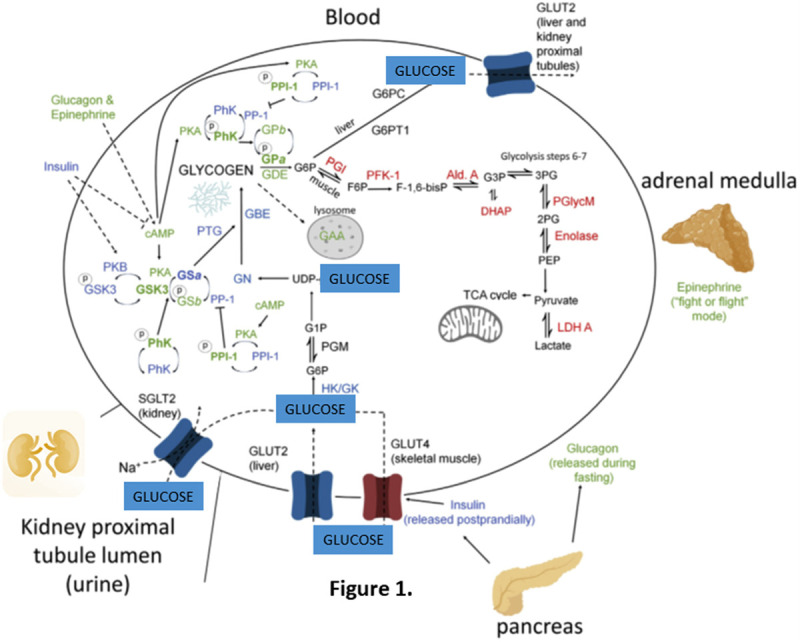
Key elements of glucose and glycogen metabolism. Enzymes in blue direct glycogen synthesis and are largely stimulated by insulin. Enzymes in green direct glycogenolysis and are largely stimulated by glucagon and epinephrine. Enzymes in red are involved in the breakdown of glucose 6-phosphate to provide energy via glycolysis. (Based on the diagram from “Glucose and glycogen in the diabetic kidney: heroes or villains? Mitchell A. Sullivan, Josephine M. Forbes” (2019)^1^).

**Figure 2:**
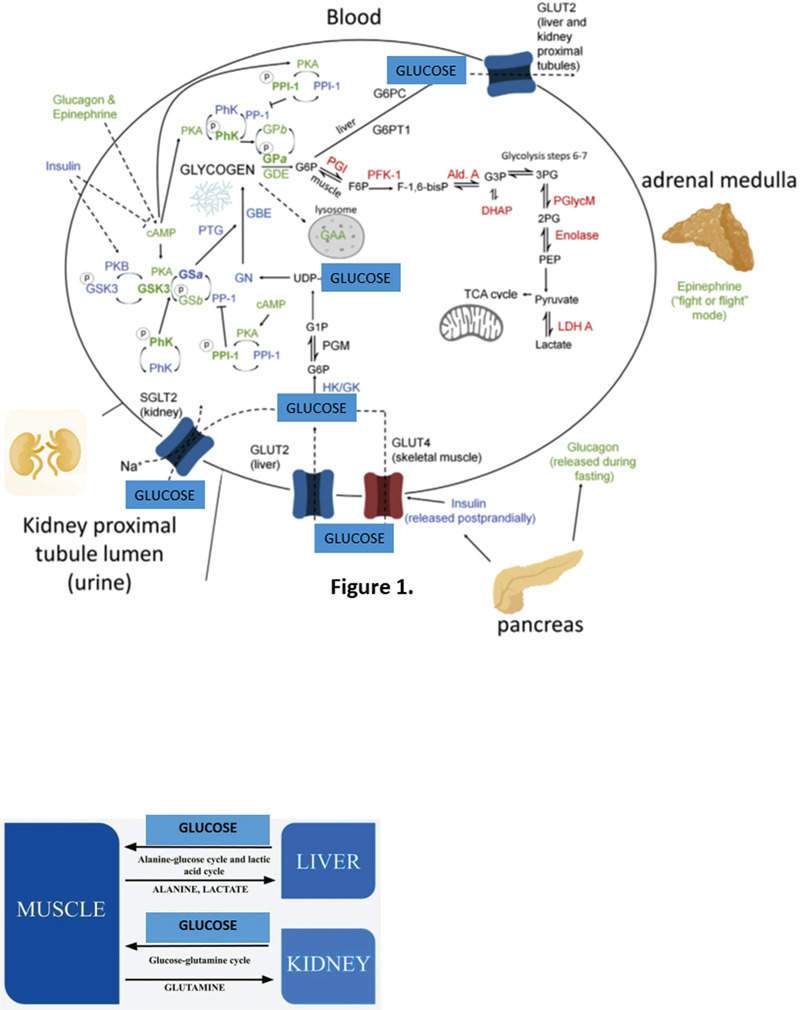
Glucose production in the process of gluconeogenesis in the liver (alanine-glucose cycle and lactic acid cycle) and in the kidney (glucose-glutamine cycle). (Based on the diagram from “Glucose Homeostasis—What Role Does the Human Kidney Play? Mitchell A. Sullivan, Josephine M. Forbes” (2020)^10^).

Glucose homeostasis is the result of the equilibrium between blood supplying glucose and consuming in cells. For the circulation pool, glucose is supplied by the digestive tract and is produced in the body in the process of gluconeogenesis. The glucose that enters the body derived from food is partially metabolized in the process of glycolysis to cover the body's current needs.^1^ It is partially stored as glycogen in the liver and muscles (glycogenesis). In the postprandial period, circulation requires a constant supply of glucose, which is an essential source of energy for tissues such as brain cells. Glucose produced in the body is another source in addition to externally supplied glucose.^1^ Only the liver and kidneys can supply glucose to the circulation through the process of gluconeogenesis, which involves the synthesis of glucose again from non-glycemic substrates; and through glycogenolysis, that is, the decomposition of stored glycogen.^1,2^ The liver is recognized as the main organ in which glucose production occurs. This process occurs in two ways. One is glycogenolysis, or degradation of hepatic glycogen, the other is gluconeogenesis, which is the synthesis of glucose from non-glycemic substrates. In physiological and pathological conditions, the kidneys play an important role in maintaining the body's metabolic homeostasis. This is due to their participation in the process of glycolysis and gluconeogenesis, in addition to two very important functions: filtration of glucose in the glomeruli and its reabsorption in the tubules.^2^ In the kidneys, glutamine is the main substrate for gluconeogenesis. The process of glucose synthesis occurs in the glucose-glutamine cycle. The process of gluconeogenesis depends on the concentration of glucose in the blood at the given time and the entry of substrates. In addition, it is subject to humoral regulation.^3,4^ Among the factors playing an essential role in the above-mentioned processes are hormones, mainly insulin and catecholamines, as well as enzymes and glucose transporters.^5–7^ The kidneys are also involved in glucose reabsorption. Sodium-glucose co-transporters—SGLT1 and SGLT2, located in segments S1 and S3 of the proximal tubule, mediate this process. The SGLT2 cotransporter^2,8^ mediates most renal glucose reabsorption (up to 90%). Glucose produced in the process of gluconeogenesis in the renal cortex is used to cover the core's energy needs. Glucose reabsorption is one of the most important physiological functions of the kidneys, enabling full recovery of filtered glucose, elimination of glucose from urine, and the prevention of calorie loss.^9^ The process of gluconeogenesis involves pyruvate carboxylase, phosphoenolpyruvate carboxykinase, fructose-1,6-bisphosphatase, and glucose-6-phosphatase, which are enzymes located mainly in kidney cortical cells^10^ (Figs. 1 and 2).

## Glucose and lipid metabolism

Glucose and lipids are both important components of energy metabolism. Maintaining a normal concentration of glucose in plasma requires an accurate match of the use of glucose and its production in daily life.^11^ Recent studies have shown that certain factors participate in glucose and lipid metabolism: TNFα, IL-6 and MCP-1,^12^ and their disregulation may associate with metabolic disorders. There is clear evidence that indicates cytokines (adipokines, hepatocins, inflammatory cytokines, myocins and osteocins) as contributors to the development of abnormal glucose and lipid metabolism, and play a positive role in metabolism action, while others have a negative metabolic role linking to the induction of metabolic dysfunction. The mechanisms involved are associated with lipid accumulation in organs and tissues, especially in the adipose and liver tissue, changes in energy metabolism, and inflammatory signals derived from various cell types, including immune cells. Given the disease-related changes in circulating levels of relevant cytokines, these factors may serve as biomarkers for the early detection of metabolic disorders.^13^

**Figure 3:**
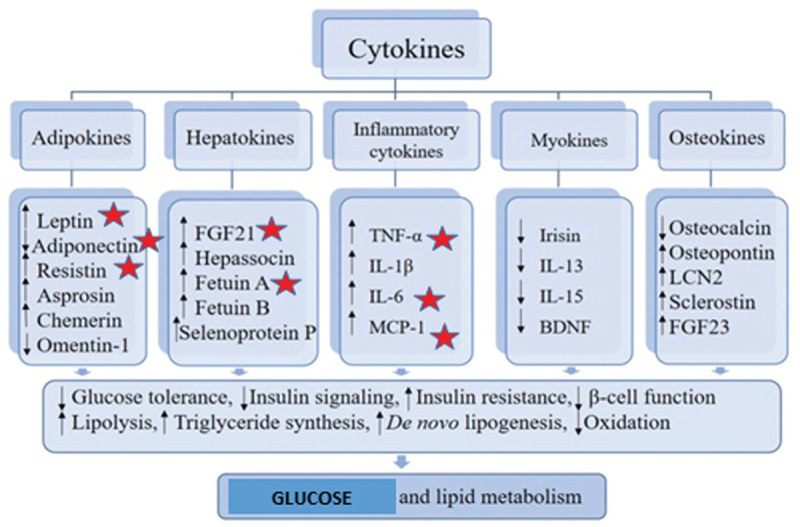
Alterations of cytokines levels and metabolic dysregulation. (Based on the diagram from “Cytokines and Abnormal Glucose and Lipid Metabolism”, Jie Shi et al (2019)^13^).

Cytokine secretion is influenced by over-nutrition and physical activity, which in turn alters the metabolic and regulatory immune pathways promoting metabolic disorders. These proteins are part of a complex network that mediates communication between multiple organs such as the liver, muscle, skeleton and, for example, adipose tissue. Due to changes in relevant cytokine levels (eg, leptin, adiponectin, reisitin, FGF21, Fetuin A, TNF-α, IL-6, MCP-1), these factors can serve as biomarkers for the early detection of metabolic disorders^13^ (Fig. 3).

## Glucose and obesity

Obesity is a growing health issue and has become a worldwide epidemic; its prevalence has been projected to grow by 40% in the next decade.^14^ This increase has implications for the risk of diabetes, cardiovascular disease, Chronic Kidney Disease, insulin resistance and various types of cancer.^14,15^

The treatment in focus for overweight and obesity is lifestyle modification, mainly a lower energy intake in combination with increased physical activity.^16^ Adjuvant pharmacotherapies typically have demonstrated modest efficacy, and this may partly be due to physiological counter-regulatory mechanisms.^17^ The SGLT2 inhibitors are glucose-lowering drugs that reduce plasma glucose levels, inhibiting glucose and sodium reabsorption in the kidneys, resulting in glycosuria. These drugs can be administrated in combination with other agents that act by decreasing food intake (eg, glucagon-like peptide 1-receptor agonists, GLP1-RAs).^18^

This is not surprising considering the fact that both, lipids and glucose play an important role in energy metabolism and are regulated by the liver.^19^ Recent human and experimental studies have associated ectopic lipid accumulation in the kidney (fat kidney) with obesity-related kidney disease. Ectopic lipid (accumulation of lipid in non-adipose tissue) is associated with structural and functional changes in mesangial cells, podocytes and proximal tubular cells to propose the development of obesity-related glomerulopathy as a maladaptive response to hyperfiltration and albuminuria.^20^

The prevalence of obesity has been accompanied by a parallel increase in disorders of glucose metabolism such as.^21^

## Glucose and inflammation

The significance of the role of inflammation in metabolic complications, particularly in diabetes, was first highlighted in a study published in 2014, when researchers found that, without inflammation, glucose could not enter and damage the body's cells.

Hyperglycemia is recognized as a pro-inflammatory condition and one of the main causes of vascular damage. Researchers have found that the addition of an inflammatory protein called “interleukin-1” to the bloodstream results in excess cell glucose metabolism, which in turn produces inflammation, even in patients with and without existing diabetes.^22^ High glucose only promotes inflammation in human vascular cells previously stimulated by pro-inflammatory stimuli, such as the interleukin (IL) 1β cytokine. A pro-inflammatory stimulus like IL1β transforms excess glucose into a deleterious vascular agent, causing an increase in glucose uptake and its subsequent diversion to pentose phosphate pathway (PPP), promoting the necessary pro-oxidant conditions for the exacerbation of pro-oxidants and pro-oxidants.^23^ Inflammatory pathways PPP overactivation is a crucial mechanism by which high glucose exacerbates damage to vascular cells. The activation of PPP by pro-inflammatory cytokines allows excess glucose to enter this metabolic pathway, creating a situation in which the formation of free radicals exceeds the cell's ability to regenerate GSH. This pro-oxidant environment increases vascular inflammation and, as a result, induces vascular damage associated with hyperglycemia.^24^

From a translational approach, anti-inflammatory treatment, associated with glucose control, is likely to be beneficial for the prevention or treatment of cardiovascular complications in diabetes. Some studies on certain compounds have shown very valid results, such as the example of the recent use of the IL-1 receptor antagonist anakinra in an animal model of diabetes,^25^ as well as by studies in patients with anakinra or canakinumab.^26^ In addition, from a therapeutic point of view, the results indicate the need not only to control blood glucose, but also to reduce inflammation, in order to avoid the potential detrimental effect of high glucose levels on vascular cells.

## Glucose in pathologies—metabolic syndrome related

The kidneys are the main organs involved in the release of insulin from the systemic circulation.^27^ They contribute to the endogenous production of glucose through gluconeogenesis, mainly in cells of the proximal tubule (PT) under glucose and insulin regulation. In addition, PTs reabsorb glucose after its glomerular filtration, through transporters linked to sodium glucose (SGLTs), mainly SGLT2 located on the luminal surface of PT cells. Consequently, renal glucose management also depends on glomerular glucose filtration and the degree of renal damage.^28,29^

The transport of glucose to mammalian cells is essential for survival. The circulating glucose in the post-absorptive state is captured by insulin-independent organs: brain (50%) and splanchnic organs (25%), with only the remainder (25%) being used in insulin-dependent tissues, especially the muscles skeletal, and, second, adipose tissue.^30,31^ However, any imbalance in this peripheral glucose uptake can lead to glucose intolerance or even diabetes mellitus. The main form of glucose entry into cells is promoted by facilitated diffusion, with the participation of specific membrane proteins, such as GLUT 1 and GLUT 4.^32,33^

The relationship between metabolic abnormalities and the development of renal disease is complex. Hyperglycemia induced complications are mediated by several metabolic pathways, among which accumulation of Advanced Glycation End-products with abnormalities of the glycosylation of macromolecules and increased glucose flux through the polyol pathway seem to be the most important. The altered glucose metabolism will ultimately result in renal functional and structural abnormalities via changes in gene expression and augmented oxidative stress leading to increased extracellular matrix production and cellular senescence.^34^

Good glycemic control has been established to delay the development of diabetic nephropathy. In the process of gluconeogenesis, approximately 50% of the body's glucose resources are generated, of which 50% are produced in the kidney. It follows that people with chronic renal failure can develop hypoglycemia.

Diabetes is a major cause of kidney failure with about 44% of end-stage renal disease worldwide.

In diabetes, the increased amount of glucose taken up by the cells is channeled into various metabolic pathways, among which the hexosamine pathway, polyol pathway, and the myoinositol pathway are the best characterized.^35^ Excess glucose is also channeled into the polyol pathway resulting in changes in the polyol-inositol metabolism important in the pathogenesis of some diabetic complications.^36^

As insulin signaling directly preserves metabolism and mitochondrial function, insulin resistance can trigger mitochondrial dysfunction and damage, contributing to kidney damage.^37^ Conversely, impaired mitochondrial function reduces insulin sensitivity.^38^

Chronic elevated plasma glucose can increase insulin resistance and dysfunction of β cells, contributing to the disturbance of glucose homeostasis. SGLT2 inhibitors are now used as the preferred medication for patients with Diabetes Mellitus Type 2 (DM2), as they reduce glucose, cause weight loss and offer protection against cardiovascular complications mainly heart failure.^38^

## Discussion and conclusions

Most of the studies included in this review relate glucose and its role in homeostasis and its relationship to metabolic syndrome. The research recognizes that new therapeutic options include SGLT2 inhibitors that block renal glucose reabsorption and can be used as monotherapy or as a supplement to oral antihyperglycemic drugs or insulin, at least in patients with DM2.^39,40,41^ Thus, it is important to know the interactions between insulin and glucose transport by PTs, to understand not only the renal impairment of diabetes, but also the interactions between therapeutic drugs, especially insulin with SGLT2i. In the presence of insulin resistance associated with DM2, glucose overproduction occurs.^38^ In recent years, much attention has also been paid to the relationship between renal failure and the degree of metabolic control in patients with type 1 diabetes (DM1), because progressive renal failure is one of the main complications of this type.^42^

Pre-clinical studies, using cytokines that can induce improvements in glucose and lipid metabolism and in the immune response may emerge as new targets for broader and more effective treatments and prevention of metabolic diseases.^13^

The available data, point to an important participation of insulin in the handling of renal glucose, including tubular glucose transport, but studies in humans with a reproducible and comparable method are still needed.^43^

In recent years, the study of the intestinal microbiota and its relationship with the CNS has revolutionized our understanding of what is thought about this large area. The prevalence of mental disorders is expected to increase in the coming years, which makes this research increasingly necessary and a complete study of the gut-brain axis will be crucial to understand its involvement in the different pathophysiology of disorders such as depression, dementia, autism or schizophrenia. However, lifestyle changes can be a good complement to reduce the metabolic and cardiovascular burden of diabetes in association with kidney disease and associated hyperglycemia.

## Conflicts of interest

The author declares that there are no conflicts of interest.
